# Comparison of the sequencing bias of currently available library preparation kits for Illumina sequencing of bacterial genomes and metagenomes

**DOI:** 10.1093/dnares/dsz017

**Published:** 2019-07-31

**Authors:** Mitsuhiko P Sato, Yoshitoshi Ogura, Keiji Nakamura, Ruriko Nishida, Yasuhiro Gotoh, Masahiro Hayashi, Junzo Hisatsune, Motoyuki Sugai, Itoh Takehiko, Tetsuya Hayashi

**Affiliations:** 1 Department of Bacteriology, Graduate School of Medical Sciences, Kyushu University, Fukuoka, Fukuoka, Japan; 2 Department of Medicine and Biosystemic Science, Graduate School of Medical Sciences, Kyushu University, Fukuoka, Fukuoka, Japan; 3 Division of Anaerobe Research, Life Science Research Center, Gifu University, Gifu, Gifu, Japan; 4 Center for Conservation of Microbial Genetic Resource, Gifu University, Gifu, Gifu, Japan; 5 Project Research Center for Nosocomial Infectious Diseases, Hiroshima University, Hiroshima, Hiroshima, Japan; 6 Department of Bacteriology, Graduate School of Biomedical and Health Sciences, Hiroshima University, Hiroshima, Hiroshima, Japan; 7 Antimicrobial Resistance Research Center, National Institute of Infectious Diseases, Tokyo, Japan; 8 Department of Biological Information, Tokyo Institute of Technology, Tokyo, Japan

**Keywords:** Illumina sequencing, sequencing bias, library preparation kits, bacterial genome sequencing, metagenome sequencing

## Abstract

In bacterial genome and metagenome sequencing, Illumina sequencers are most frequently used due to their high throughput capacity, and multiple library preparation kits have been developed for Illumina platforms. Here, we systematically analysed and compared the sequencing bias generated by currently available library preparation kits for Illumina sequencing. Our analyses revealed that a strong sequencing bias is introduced in low-GC regions by the Nextera XT kit. The level of bias introduced is dependent on the level of GC content; stronger bias is generated as the GC content decreases. Other analysed kits did not introduce this strong sequencing bias. The GC content-associated sequencing bias introduced by Nextera XT was more remarkable in metagenome sequencing of a mock bacterial community and seriously affected estimation of the relative abundance of low-GC species. The results of our analyses highlight the importance of selecting proper library preparation kits according to the purposes and targets of sequencing, particularly in metagenome sequencing, where a wide range of microbial species with various degrees of GC content is present. Our data also indicate that special attention should be paid to which library preparation kit was used when analysing and interpreting publicly available metagenomic data.

## 1. Introduction

Next-generation sequencing (NGS) has revolutionized the field of genomics,^1^^,^^2^ as it has much higher throughput (thus much lower cost) compared with traditional Sanger sequencing.^3^ The use of Illumina sequencing technology dominates the fields of bacterial genomics and metagenomics. Therein, library construction is an important process. While several library construction methods have been developed for Illumina sequencing,^4^^,^^5^ this process generally comprises three steps: DNA fragmentation, repairing and end-polishing of fragmented DNA, and ligation of platform-specific adaptors.^6^ Several library preparation kits are now commercially available, and they employ sonication or enzymatic digestion for DNA fragmentation. Nextera XT DNA Library Preparation Kit (XT), which employs a transposon to shear genomic DNA and simultaneously introduce adapter sequences,^7^ is an alternative approach for streamlining the workflow, improving turnaround time and reducing DNA input.

In Illumina sequencing, extreme base composition, i.e. extremely GC-poor or rich sequences, has been reported to yield uneven or poor sequencing coverage.^8–10^ For example, Illumina sequencing of *Plasmodium falciparum* (mean GC; 19%) and *Rhodobacter sphaeroides* (69%) favoured more GC-balanced regions and yielded fewer reads from regions of GC content at either extreme.^11^ Such a sequencing bias reduces the efficacy of data analyses; genome assembly,^12^^,^^13^ identification of sequence variations by read mapping,^14^^,^^15^ and estimation of the copy numbers of sequences of interests. Lower-coverage regions may lead to a failure to identify single-nucleotide polymorphisms and genomic regions of functional or phylogenetical importance. Efforts to reduce gaps or low-coverage regions by obtaining more sequence reads inflate sequencing costs and may limit the effectiveness of genomic analyses, particularly, those aiming to analyse numerous samples. Thus, improving our knowledge of sequencing bias is essential to further improve the utility of sequencing by NGS.

Uneven coverage associated with GC bias can be introduced during PCR amplification of library, cluster amplification, or sequencing. Among these, library amplification is known as a major source.^16^^,^^17^ The XT kit has been reported to introduce a substantial sequencing bias in *Mycobacterium tuberculosis* sequencing (mean GC; 66%)^18^ and yielded more unmapped open reading frames for *Clostridium beijerinckii* (30%) in a mock metagenome sample compared with other kits.^19^ We also found inefficiency issues in assembling *Staphylococcus aureus* genomes (33%) from Illumina reads obtained by XT. Because studies on sequencing bias have been conducted in limited strains or species, systematic investigation of bacteria with a wide GC content range is required to understand the factors that introduce sequencing bias. Different sequencing kits and protocols may also be differentially affected by GC bias. The resulting sequence bias also impairs interpretation of metagenome sequencing data, which include many unknown species, particularly in estimating the relative abundances of genes/species in a microbial community based on read counting. Therefore, in this study, we compared currently available library preparation kits for Illumina sequencing, including the Nextera DNA Flex Library Prep Kit recently released by Illumina, to examine what kinds and what levels of sequencing bias are generated by these kits across a wide range of bacterial species. The impacts of sequencing bias on metagenome analysis were also evaluated using a mock bacterial community comprising species with a wide range of GC content levels.

## 2. Materials and methods

### 2.1. Strains and genomic DNA preparation

We used two *Escherichia coli* strains, K-12 MG1655 and O157 Sakai, and two *S. aureus* strains, MW2 and N315, as model microbial organisms to analyse sequencing bias introduced by different library preparation kits (Supplementary Table S1). The complete genome sequences of these strains are available.^20–23^ In addition, 22 strains of non-*S. aureus* species in the genus *Staphylococcus* and seven species with various degrees of GC content from the strain collections of our laboratories were analysed (Supplementary Table S1). The *E. coli* strains were grown in Lysogeny broth (LB; Becton Dickinson Microbiology Systems, MD, USA) at 37°C. Genomic DNA was purified from 2 ml of overnight culture using a Genomic-tip 100/G and Genomic DNA buffer set (QIAGEN, CA, USA) according to the manufacturer’s instructions. *Staphylococcal* strains were grown at 37°C overnight with shaking in 2-ml tryptic soy broth (Becton Dickinson Microbiology Systems), and genomic DNA was isolated as previously described.^24^ Briefly, the cells were collected by centrifugation, resuspended in 500 µl of CS buffer (100 mM Tris-HCl pH 7.5, 150 mM NaCl, 10 mM EDTA), incubated sequentially with 20 µg/ml lysostaphin (Wako, Tokyo, Japan) for 30 min at 37°C and with 100 µg/ml Proteinase K and 1% SDS (both from Nacalai Tesque, Kyoto, Japan) at 55°C for 2 h, and then subjected to phenol-chloroform extraction and ethanol precipitation. The genomic DNA was finally cleaned up using a Genomic DNA Clean & Concentrator kit (Zymo Research, CA, USA). Among the seven species with various degrees of GC content, *M. interjectum* and *M. malmoense* were grown in Middlebrook 7H9 broth (Becton Dickinson Microbiology Systems) with albumin dextrose catalase enrichment. *Tsukamurella pulmonis*, *Klebsiella aerogenes*, *Streptobacillus moniliformis*, and *Brachyspira pilosicoli* were grown on sheep blood agar at 37°C, and *Serratia liquefaciens* was grown on LB agar at 37°C. Genomic DNA from these bacteria was prepared using a NucleoSpin Microbial DNA kit (Takara Bio Inc., Shiga, Japan).

A mock microbial community DNA was composed of genomic DNA from *E. coli* MG1655, *S. aureus* N315, *M. interjectum*, *M. malmoense*, *T. pulmonis*, *K. aerogenes*, *S. liquefaciens*, *S. moniliformis*, and *B. pilosicoli*, representing an approximately equimolar mixture of these genomic DNAs.

### 2.2. Genome sequences obtained from a public database

The genome sequencing data of 204 human pathogenic bacterial species were obtained from the DDBJ Sequence Read Archive database in fastq format. These strains were from the Gifu Type Culture (GTC) collection of Gifu University Center for Conservation of Microbial Genetic Resource (GCMR) and were sequenced by the National BioResource Project (NBRP) of Japan using an XT library preparation kit (Illumina, CA, USA) and the Illumina HiSeq 2500 platform (Supplementary Table S1).

### 2.3. Library preparation and sequencing

Eight sequencing library preparation kits were used in this study: XT, KAPA HyperPlus (NIPPON Genetics Co. Ltd, Tokyo, Japan) with PCR or PCR-free workflow (referred to as KP and KPF, respectively), NEBNext Ultra II (referred to as NN; New England Biolabs Japan, Tokyo, Japan), QIAseq FX (QS; QIAGEN), TruSeq nano (TS; Illumina), TruSeq DNA PCR-Free (TSF; Illumina), and Nextera DNA Flex (FL; Illumina) (Table 1). For XT, KP, NN, QS, and FL, 1 ng of DNA, measured by Agilent TapeStation (Agilent Technologies, CA, USA), was used for input DNA, and the number of PCR cycles was fixed at 12. These parameters were set according to the XT protocol, for which the amount of input DNA and the number of PCR cycles were essentially unable to be modified. For the TS library preparation, 200 ng of input DNA and eight PCR cycles were employed. For KPF and TSF, 1 µg of input DNA was used. Other procedures for library preparation were performed according to the recommended protocols of each kit. The prepared libraries were sequenced on the Illumina MiSeq platform with a paired-end (PE) 600-cycle mode (Reagent Kit v3). The XT libraries of the *E. coli* and *S. aureus* genomes were prepared independently three times from the same genomic DNA preparation to generate technical replicates. The sequencing reads have been deposited in DDBJ (accession number: PRJDB8030).

**Table 1:** Library preparation kits analysed in this study

Kits	Abbreviation	Fragmentation methods	PCR cycles	Input DNA (ng)
Nextera XT	XT	Tagmentation by transposome	12	1
Nextera DNA Flex	FL	Tagmentation by transposome	12	1
KAPA HyperPlus	KP	Enzymatic	12	1
NEBNext Ultra II	NN	Enzymatic	12	1
QIAseq FX	QS	Enzymatic	12	1
TruSeq nano	TS	Sonication	8	200
KAPA HyperPlus PCR-free workflow	KPF	Enzymatic	0	1,000
TruSeq DNA PCR-free	TSF	Sonication	0	1,000

### 2.4. Genome assembly

PCR duplicates were removed by FastUniq,^25^ and adapter and low-quality sequences were trimmed by trim galore! v0.4.2 (http://www.bioinformatics.babraham.ac.uk/projects/trim_galore/) for all sequencing data. Trimmed reads from each sample were assembled using Velvet v1.2.10 and VelvetOptimiser v2.2.5^12^ with k-mer lengths ranging from 19 to 199. Contigs shorter than 300 bp were excluded from this study. To compare the numbers of contigs obtained by the six library preparation kits under the same conditions (e.g. at the same sequencing depth), sequencing reads were randomly picked up from each of the four model genomes (two *E. coli* and two *S. aureus* genomes) to gain ×30 coverage, which was calculated based on the total genome length of each strain. This procedure was repeated ten times for each genome. The four model genomes were also assembled by SPAdes v3.9.0^13^ with default parameters.

### 2.5. Sequencing bias analysis

Insert sizes of each library were calculated using Picard tools version 2.7.0 (http://broadinstitute.github.io/picard/), and jellyfish v2.2.6^26^ was used to calculate the 30-mer frequency. Per-base coverage was computed by counting the reads mapped to a given base along the reference genome.^11^ We used bowtie2^14^ and SAMtools^27^ for read mapping and counting the coverage of each base, respectively. As references, the published complete sequences were used for the four model genomes from *E. coli* and *S. aureus*, while draft sequences were used for others. The draft sequences were generated by merging and assembling sequencing reads obtained by all kits in each strain, and contigs shorter than N90 were excluded from the references because they might be derived from multicopy genetic elements. The qualities of the assemblies from 204 strains obtained from the NBRP data were assessed by checkM,^28^ and 13 genomes with low completeness (<85%) or high contamination (>5%) were excluded from this study (final *n* = 191).

To evaluate coverage bias (a deviation from the uniform distribution of reads across the genome), we first calculated per-base relative coverage, which is the ratio of the coverage of a given reference base to the mean coverage across the genome.^11^ Then, ‘relative coverage’ was presented as the mean per-base relative coverage of each 200-bp window with no overlap.

To evaluate the level of sequencing bias associated with various degrees of GC content, GC content was calculated for each 200-bp window. The 200 bp windows with similar GC content levels (defined by a 0.5% interval) were binned, and the mean relative coverage of each bin was calculated; here, the mean relative coverage of the bin with a given GC content was represented as $C_{\mathrm{GC}}$. Finally, to quantify the overall sequencing bias associated with GC content in a given genome, the overall GC content-associated bias was defined as:

$$\frac{\sum_{i}n_{i}\sqrt{{\left(C_{i}-C\right)}^{2}}}{n_{T}N}$$

where *i* is GC content defined at 0.5% intervals; $C_{i}$ is the $C_{\mathrm{GC}}$ of the bin with *i* % GC; *C* is the mean relative coverage across the genome (=1); $n_{i}$ is the number of windows with *i* % GC; $n_{T}$ is the total number of windows; and *N* is the total number of analysed bins. We used linear regression models to investigate the relationship between the overall GC content-associated sequencing bias and mean GC content across the genome using R v3.2.4.

### 2.6. Digital droplet PCR and mock community analysis

Accurate molecular ratios of the seven species in the pooled genomic DNA sample for the mock microbial community were determined by digital droplet PCR (ddPCR) using ddPCR™ EvaGreen Supermix (BioRad, CA, USA) and species-specific primers (Supplementary Table S2), which were designed in the arginyl-tRNA synthetase gene, one of the universal single-copy genes. Droplets were generated using a QX200™ droplet generator (BioRad), and each ddPCR sample was composed of sample DNA, primers, and ddPCR super mix. PCR was performed with the following conditions: 95°C for 5 min, 40 cycles of 94°C for 30 s, and 58°C for 90 s, and a final incubation at 90°C for 5 min. The data were analysed by QuantaSoft version 1.7.4 (BioRad). The established copy numbers of each genome in the pooled DNA sample were used to normalize the relative abundance of genomic DNA and per-base coverage for each species within the mock microbial community.

## 3. Results and discussion

### 3.1. Comparison of six library preparation kits using *E. coli* and *S. aureus* as model bacterial genomes

We first used two *E. coli* and two *S. aureus* genomes as model genomes for comparing the quality of the libraries prepared by different library preparation kits. The six kits compared in this study cover three fragmentation strategies (Table 1): tagmentation by transposome (XT and FL), enzymatic fragmentation (KP, NN, and QS), and sonication (TS). FL utilizes improved tagmentation chemistry to obtain a uniform fragment size; KP, NN, and QS each use different enzymes for fragmentation.

**Figure 1:**
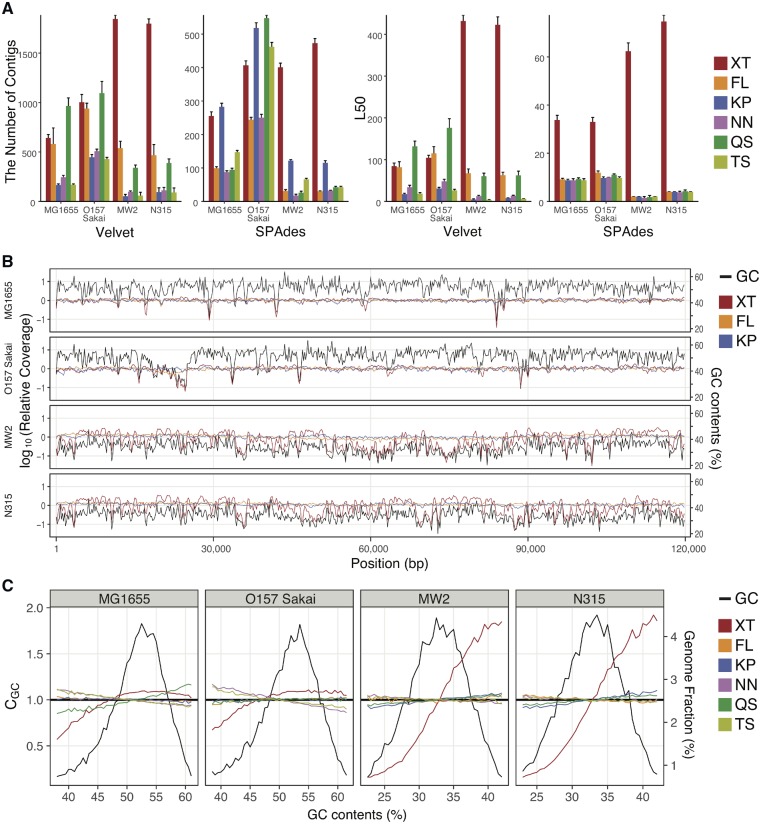
Quality comparison of *E. coli* and *S. aureus* genome assemblies obtained by library preparation kits. (A) Assembly statistics obtained by six library preparation kits were compared in *E. coli* and *S. aureus*. Two *E. coli* and two *S. aureus* genomes were analysed as model bacterial genomes to compare six library preparation kits. Illumina read sequences obtained from each library were assembled using Velvet and SPAdes, and the numbers of contigs and L50 values of each assembly are shown. In each sequence data set, assembly was repeated 10 times using Illumina reads randomly selected at 30× coverage. Error bars indicate standard deviations. The six kits used cover three fragmentation strategies (see the main text). XT, Nextera XT; FL, Nextera DNA Flex; KP, KAPA HyperPlus; NN, NEBNext Ultra II; QS, QIAseq FX; and TS, TruSeq nano. (B) Relative sequence coverage in relation to GC content was calculated in *E. coli* and *S. aureus* genomes obtained by three library preparation kits. Relative sequence coverage in the genome assemblies obtained by the XT, FL, and KP kits and GC content were calculated for every 200-bp window with no overlap. Only the first 120,000 bp regions of each genome are shown. (C) Relationships between GC content and sequence coverage in the *E. coli* and *S. aureus* genome assemblies obtained by six library preparation kits are shown. The relative abundance of 200 bp bins with a given GC content (defined by 0.5% interval) and the mean relative coverage of bins with a given GC content ($C_{\mathrm{GC}}$) were calculated and are shown along with GC content by black lines or lines coloured according to the library preparation kits, respectively. Black horizontal lines ($C_{\mathrm{GC}}$=1) represent unbiased coverage. The data for bins with extreme GC content (those representing <0.5% of all 200 bp bins) are not shown. Color figures are available at *DNARES* online.

To assess the difference in the efficacy of sequence assembly between the kits, we first compared the number of contigs and L50 values of each assembly obtained by Velvet and SPAdes at the same sequencing depth (×30) (Fig. 1A). In *E. coli*, the number of contigs and L50 values exhibited some level of variation between the kits and between the strains and assemblers used. In contrast, the levels of sequence assembly of both *S. aureus* genomes obtained by XT were much lower than those obtained by other kits, regardless of which assembler was used. Analysis of k-mer frequencies in each read data set revealed that a sharp peak was observed in *S. aureus* data sets with the exception of the data set obtained by XT (Supplementary Fig. S1). This result suggests that the inefficient sequence assembly observed for the XT-derived *S. aureus* assemblies was caused by uneven genome sequencing. In fact, sequence coverage across the genome in each library assessed by calculating the relative coverage in every 200-bp window (see Materials and methods) revealed that the XT-derived assemblies of *S. aureus* genomes showed remarkably uneven coverage along the entire genome (Supplementary Fig. S2). Notable bias was also observed in the XT-derived assemblies of *E. coli* genomes but to a much lesser extent than in the *S. aureus* genomes.

Because sequencing bias associated with GC content has been reported for Illumina sequencing,^8–10^ we next investigated the relationship between relative coverage and GC content across the *E. coli* and *S. aureus* genomes (Fig. 1B). The results of this analysis clearly indicated that the variations in relative coverage in the XT libraries correlated well with the variations in GC content, particularly in the *S. aureus* genomes, with lower coverage in regions with lower GC content. In Fig. 1B, only the data for a 120-kb region in each genome are shown, but similar patterns were observed along the entire genome (data not shown). To quantitatively evaluate the level of GC content-associated sequencing bias at the whole-genome level, we calculated $C_{\mathrm{GC}}$, which represents the mean relative coverage of a 200-bp bin with a given GC content (see Materials and methods for more details), across the genome and analysed the GC content-associated variation in $C_{\mathrm{GC}}$ in each library (Fig. 1C). This analysis revealed that in the XT libraries of *S. aureus*, $C_{\mathrm{GC}}$ was drastically reduced as GC content decreased. A reduction in $C_{\mathrm{GC}}$ was also observed in the XT libraries of *E. coli*, but only in the bins with lower GC content. In contrast, similar $C_{\mathrm{GC}}$ values were observed in bins of all GC content values in the libraries prepared with the other five kits, including FL. We prepared XT libraries three times from all *E. coli* and *S. aureus* genomes and performed the same analyses as technical replicates. Although the results of *E. coli* libraries showed some variation between the samples, the results of *S. aureus* genomes were highly reproducible (Supplementary Fig. S3).

All these results indicate that a notable GC content-associated sequencing bias in Illumina sequencing is introduced by XT, particularly in *S. aureus* genome sequencing.

### 3.2. Sequencing bias in 22 strains from various species in the genus *Staphylococcus*

To examine whether the GC content-associated sequencing bias observed for XT is species-specific, we assessed the sequencing bias introduced by XT in other *Staphylococcus* species. We analysed 22 strains of 15 non-*S. aureus* species in the genus *Staphylococcus* (Supplementary Table S1). KP was used as a control kit. In all strains analysed, while $C_{\mathrm{GC}}$ values in the KP libraries were nearly even regardless of the GC content (Supplementary Fig. S4), $C_{\mathrm{GC}}\;$in the XT libraries drastically decreased as GC content decreased, as seen in *S. aureus*.

**Figure 2:**
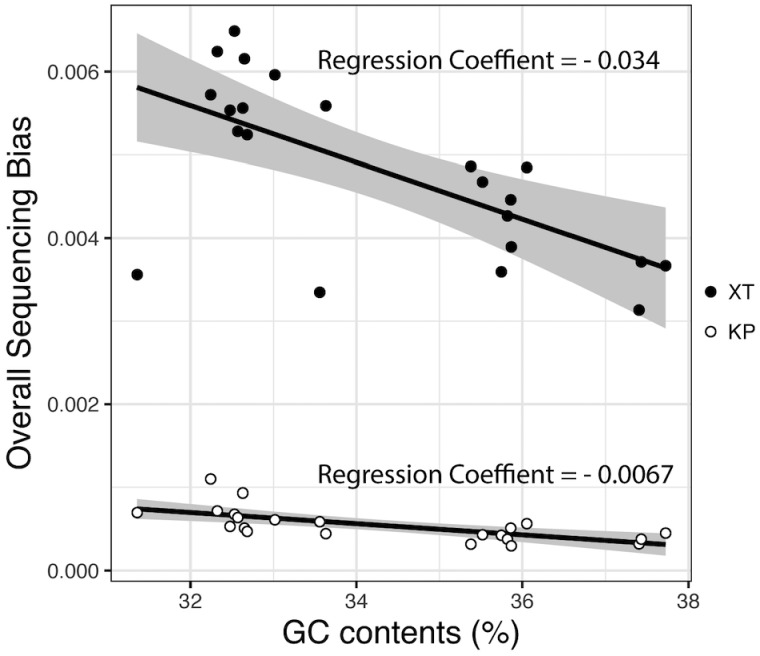
Overall GC content-associated sequencing bias observed in 22 strains of non-*S. aureus* species in the genus *Staphylococcus*. Sequence reads were obtained from 22 strains of non-*S. aureus* species in the genus *Staphylococcus* using the XT and KP kits. The overall sequencing bias associated with GC content observed in the genome assemblies was quantified (see Materials and methods in the main text), and the relationships between the quantified overall sequencing bias and the mean GC content of each genome are shown. Solid lines indicate regression lines, and the 95% confidence intervals are indicated in grey.

We quantified the overall GC content-associated sequencing bias by calculating the average deviation of $C_{\mathrm{GC}}$ (see Materials and methods) and performed a linear regression analysis of the relationship between overall GC content-associated bias and mean GC content of each genome (Fig. 2). These analyses revealed that, in all strains, the overall sequencing bias was much higher in XT libraries than in KP libraries. Moreover, the levels of overall sequencing bias correlated well with the mean GC content of genomes, particularly in the XT libraries; the regression coefficients of XT and KP were −0.034 (*P* value = 0.0010) and −0.0067 (*P* value = 0.0005), respectively. These results indicated that the strong GC content-associated sequencing bias introduced by XT is common to the members of the genus *Staphylococcus*.

### 3.3. Sequencing bias in 191 species representing a wide range of bacterial species

**Figure 3:**
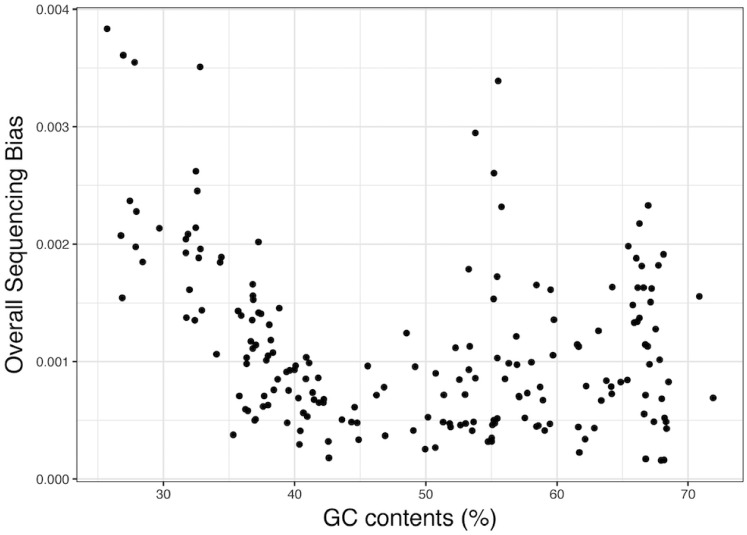
Overall GC content-associated sequencing bias in the sequence data of 191 species obtained by the XT library preparation kit. Illumina sequencing data for 191 species (one strain from each species) produced using the XT kit from a project of NBRP of Japan were downloaded from the public database (DDBJ). The overall GC content-associated sequencing bias in each data set was quantified, and relationships between the quantified overall sequencing bias and the mean GC content of each genome are shown.

We further investigated the overall GC content-associated sequencing bias introduced by XT across a wide range of bacterial species using 191 genomes from various species in the GTC collection (one strain from each species), which were sequenced by NBRP of Japan. These species were from Proteobacteria (*n* = 90), Actinobacteria (*n* = 40), Bacteroidetes (*n* = 11), Firmicutes (*n* = 47), and three other phyla (*n* = 3) and had a wide range of genome sizes (1.5–6.4 Mb) and GC content (25.7–71.9%) (Supplementary Table S1). As shown in Fig. 3 (see Supplementary Fig. S5 for the data of each genome), although overall sequencing bias was observed in many species, a stronger bias was more frequently observed in the species with an extreme mean GC content. Particularly in low-GC species (<40%), stronger bias was observed in all genomes, and the levels of bias correlated with the levels of GC content, as seen in the genus *Staphylococcus*. In many species with higher GC content, particularly those with extremely high GC content (>65%), high levels of overall sequencing bias were also observed. In most of these species, $C_{\mathrm{GC}}$ gradually decreased as the GC content increased, as opposed to the trend in low-GC species (see Supplementary Fig. S5). The results of this analysis indicated that XT introduces GC content-associated sequencing bias in a wide range of bacterial species, particularly those with extreme GC content, although sequencing bias in lower and higher GC regions may not be caused by the same mechanism.

### 3.4. Comparison of sequencing bias between the methods employing different fragmentation and amplification techniques

To confirm the reproducibility of sequencing bias introduced by XT and to evaluate the effects of PCR amplification, we prepared genomic DNA from seven species that showed high levels of sequence bias in the analyses described above and prepared their sequencing libraries using four library preparation kits utilizing various fragmentation methods (XT, FL, KP, and TS) and two PCR-free kits (KPF and TSF). The seven analysed species were two low-GC species (*B. pilosicoli* and *S. moniliformis*), two intermediate-GC species (*S. liquefaciens* and *K. aerogenes*), and three high-GC species (*M. malmoense*, *M. interjectum*, and *T. pulmonis*). Analyses of the sequences obtained from each library revealed that strong GC content-associated sequencing bias was reproducibly observed in the XT libraries of the two low-GC species (Supplementary Fig. S6). However, strong bias was not observed in the XT libraries of the two intermediate-GC species, although some bias was introduced in low-GC regions. In the three high-GC species, some levels of GC content-associated sequencing bias were detected in the XT libraries, but the observed bias was not as strong as that in the data set produced by NBRP of Japan. Although the reason(s) for the stronger bias observed in the NBRP data set is unknown, several factors, such as the accuracy of quantification of input DNA and lengths of input DNA, might have affected the quality of these libraries prepared with XT.

In the libraries prepared with other kits, including the recently released FL kit by Illumina, no strong bias was observed. Some GC content-associated sequencing bias was observed in the TS libraries of the two low-GC species (Supplementary Fig. S6), but no such bias was detected in the libraries prepared with TSF (PCR-free). This finding suggests that PCR amplification during library preparation can also introduce some levels of bias in low-GC regions. However, the effects are not as strong and are unlikely to cause serious problems in bacterial genome sequencing.

### 3.5. Analysis of the mock microbial community

**Figure 4:**
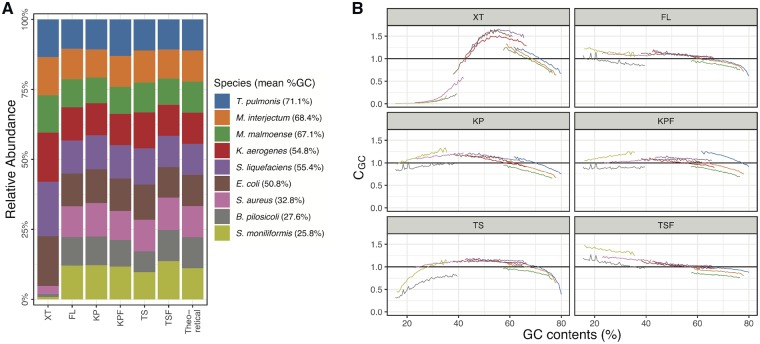
Metagenome sequencing of a mock bacterial community using six library preparation kits and the sequencing bias introduced by each kit. (A) Libraries of a mock bacterial community prepared by six library preparation kits were sequenced, and the relative genome abundance estimated in each data set obtained by six library preparation kits is shown. The mock community was composed of nine species with various levels of GC content. The relative abundances of each species were normalized by their genome sizes and the copy numbers of each species in the sample, which were determined by ddPCR. (B) Relationships between the GC content and sequence coverage in each genome in the mock community are shown. The mean relative coverage of each 200-bp bin with a given GC content ($C_{\mathrm{GC}}$) in each genome was calculated in each data set and is shown according to GC content by coloured lines. The colours of the lines correspond to the species shown in panel (A). Black horizontal lines in each plot ($C_{\mathrm{GC}}$=1) represent unbiased coverage. The relative coverage was normalized by the copy numbers in the sample determined by ddPCR. Data for bins with extreme GC content (those representing <0.5% of all 200 bp bins) are not shown. Color figures are available at *DNARES* online.

GC content-associated sequencing bias could be a more serious problem in metagenomics, where a wide range of microbial species with various degrees of GC content is sequenced. Therefore, we analysed the bias introduced in metagenome sequencing by XT and other kits (FL, KP, KPF, TS, and TSF) using a mock bacterial community DNA sample. The sample was composed of genomic DNA from the following nine bacteria: *S. aureus* (strain N315), *E. coli* (strain MG1655), and the seven species used in the analysis described above (Fig. 4). In the relative abundance analysis of the nine bacteria (Fig. 4A), the abundance of three low-GC species, *S. aureus*, *B. pilosicoli*, and *S. moniliformis*, in the XT library was remarkably lower than the expected values, which were calculated and normalized based on their molecular ratios in the sample examined. Furthermore, in relative coverage analysis within each genome, strong GC content-associated sequencing bias was evident not only in the three low-GC species but also in other species (Fig. 4B).

Other kits, including FL, did not introduce the strong bias observed in the XT library. However, a notable bias was observed in the TS library at regions with extreme GC content. The bias introduced by TS was reproducibly detected in a separate experiment (Supplementary Fig. S7). Such a bias was not observed in the library prepared with TSF, which also employs sonication for fragmentation but does not include PCR amplification. This finding suggests that the bias observed in the TS libraries may be generated by a combination of some level of non-random DNA fragmentation during sonication and PCR amplification.

### 3.6. Possible mechanisms to generate sequencing bias

The main mechanism to generate sequencing bias in XT libraries is probably uneven tagmentation affected by local GC-content, particularly AT-rich sequences, because the GC content-associated insertion bias of Tn5 transposase has been pointed out by several studies.^29–31^ It appears that less frequent insertion in GC-unbalanced regions than in balanced regions. As AT-richness increases, higher level of sequencing bias was observed in our data set. This observation suggests the gradual decrease in insertion frequency of Tn5 transposase according to the level of AT-richness, which resulted in the increase in sequencing bias in the genomes or genomic regions of bacterial strains with more extreme mean GC-contents as observed in the strains with <40% GC content. No clear difference was observed between KP and KPF libraries. However, it appears that PCR amplification during library preparation also has some contribution to bias generation because some bias was observed in the TS libraries of low GC species but not in their TSF libraries (Supplementary Fig. S6). When single genomes were analysed, the effect was not so prominent, and practically not problematic. However, in the analysis of bacterial community DNA, significant bias was introduced by TS but not TSF. This bias observed in the TS library may be generated by a combination of some level of non-random DNA fragmentation during sonication and PCR amplification. Importantly, the problem of strong sequencing bias due to uneven tagmentation has been solved in FL by employing bead-linked transposases and by some other modifications,^32^ although it is not open to users whether XT and FL use the same transposase or FL uses an improved enzyme.^31^

## 4. Conclusion

By systematic comparison of currently available library preparation kits for Illumina sequencing, we demonstrated that strong sequencing bias is introduced in low-GC regions by the Nextera XT kit. The level of bias introduced is dependent on the level of GC content; stronger bias is generated as the GC content decreases. More substantial GC content-associated sequencing bias was introduced by Nextera XT in metagenome sequencing of a mock bacterial community. Other kits, including the Nextera DNA Flex Library Prep Kit, a recently released kit from Illumina, did not introduce strong GC content-associated sequencing bias, but the TruSeq nano kit generated notable bias in regions with extreme GC content when used for metagenome sequencing. Our data indicate the importance of selecting proper library preparation kits according to the purposes and targets of genome sequencing, particularly sequencing of low-GC species and metagenome sequencing. Special attention should also be paid to which library preparation kit was used when analysing and interpreting publicly available data.

## Supplementary Material

dsz017_Supplementary_Data
