# Supplementary material for: Comparison of the sequencing bias of currently available library preparation kits for Illumina sequencing of bacterial genomes and metagenomes
Source: DNA Res. 2019 Jul 31;26(5):391–8. doi: 10.1093/dnares/dsz017 (PMC6796507; doi:10.1093/dnares/dsz017)
Supplement: dsz017_Supplementary_Data [file dsz017_supplementary_data.zip › dsz017-Suupl_data/Supplementary_Figure.doc]

**Supporting information**

**Supplementary Table S1. List of strains used in this study.**

**Supplementary Table S2. Primer sequences and concentrations for ddPCR.**

**Supplementary Fig. S1. 30-mer frequencies in the sequencing reads obtained by six library preparation kits.**

In each read set obtained by six library preparation kits (corresponding to the sets in Fig. 1 in the main text), reads were randomly selected 10 times to gain 30x coverage, and 30-mer frequency was calculated in each set of selected reads. Data for the 30-mer sequences that appeared only once or twice and those that appeared more than 50 times are not shown.

**Supplementary Fig. S2. Relative coverage along the genome in the sequencing read sets obtained by each of the six library preparation kits.**

Relative coverage in the read sets obtained by each library preparation kit was calculated for every 200-bp window with no overlap along the entire genome. Black horizontal lines (coverage = 1) represent unbiased coverage.

**Supplementary Fig. S3. Reproducibility of sequencing bias introduced by the XT library preparation kit.**

Libraries were prepared three times using the XT kit (XT1, XT2, and XT3) for the four genomes, and Illumina reads were obtained from each library.

(Top) The numbers of contigs and L50 values in each read set. In each library, assembly was repeated 10 times using Illumina reads randomly selected at 30x coverage. Two assemblers, Velvet and SPAdes, were used. Error bars indicate standard deviations.

(Middle) The 30-mer frequency in each read set. Reads were randomly selected 10 times to gain 30x coverage, and 30-mer frequency was calculated in each set of selected reads. Data for the 30-mer sequences that appeared only once or twice and those that appeared more than 50 times are not shown.

(Bottom) Relationships between GC content and sequence coverage. The relative abundance of 200-bp bins at a given GC content (defined by 0.5% interval) and the mean relative coverage of each bin with a given GC content (*C_GC_*) were calculated and are shown according to GC content by black lines and coloured lines, respectively. Black horizontal lines (*C_GC_* =1) represent unbiased coverage. The data for bins with extreme GC content (those representing less than 0.5% of all 200-bp bins) are not shown. XT1 corresponds to XT in Fig. 1 and Fig. 3 in the main text and Supplementary Fig. S1.

**Supplementary Fig. S4. Relationships between GC content and sequence coverage in 22 strains of non-*S. aureus* species in the genus *Staphylococcus*.**

Sequence reads were obtained from 22 strains of non-*S. aureus* species in the genus *Staphylococcus* using XT and KP kits. The relative abundance of 200-bp bins at a given GC content (defined by 0.5% interval) and the mean relative coverage of each bin with a given GC content (*C_GC_*) were calculated and shown according to GC content by black lines and coloured lines, respectively. Strains were ordered according to the mean GC content of genomes. The data for bins with extreme GC content (those representing less than 0.5% of all 200-bp bins) are not shown.

**Supplementary Fig. S5. Relationships between GC content and sequence coverage in the sequencing data for 191 species obtained by the XT library preparation kit.**

Illumina sequencing data of 191 species (one strain from each species) produced using the XT kit in a project of NBRP, Japan, were downloaded from the public database (DDBJ). The mean relative coverage of each bin with a given GC content (*C_GC_*) was calculated in each data set and is shown according to GC content by red lines. Species were ordered according to the mean GC content of genomes. Black horizontal lines (*C_GC_* =1) represent unbiased coverage. Data for bins with extreme GC content (those representing less than 0.5% of all 200-bp bins) are not shown.

**Supplementary Fig. S6. Relationships between GC content and sequencing coverage in the sequencing data of seven species obtained by six library preparation kits.**

Seven species, in which highly biased sequencing coverage was introduced by the XT kit, were selected from the 191 species analysed in Fig. 5 in the main text. Sequence reads were obtained from each species using six library preparation kits. The relative abundance of 200-bp bins at a given GC content (defined by 0.5% interval) and the mean relative coverage of each bin with a given GC content (*C_GC_*) in each data set were calculated and are shown according to GC content by black lines and coloured lines, respectively. Black horizontal lines (*C_GC_* =1) represent unbiased coverage. The data for bins with extreme GC content (those representing less than 0.5% of all 200-bp bins) are not shown.

**Supplementary Fig. S7. Reproducibility of the GC content-associated sequencing bias introduced in the mock community sequence data by the TS kit.**

Libraries were prepared twice from the mock bacterial community analysed in Fig. 6 (TS1 and TS2) in the main text using TS, and Illumina reads were obtained from each library. The mean relative coverage of each bin with a given GC content (*C_GC_* in each genome was calculated in each data set and is shown according to GC content by coloured lines. The relative coverage was normalized by the copy numbers in the sample determined by ddPCR. Black horizontal lines (*C_GC_* =1) represent unbiased coverage. TS1 corresponds to the TS sample in Fig. 6 in the main text.
